# Addressing *Cryptosporidium* Infection among Young Children in Low-Income Settings: The Crucial Role of New and Existing Drugs for Reducing Morbidity and Mortality

**DOI:** 10.1371/journal.pntd.0004242

**Published:** 2016-01-28

**Authors:** David A. Shoultz, Eugenio L. de Hostos, Robert K. M. Choy

**Affiliations:** PATH, San Francisco, California, United States of America; Oxford University Clinical Research Unit, VIET NAM

Diarrheal disease is the world’s second leading cause of death among young children. It claims more than half a million lives of children under five years old each year, accounting for 9% of global child deaths [1,2]. It is also a leading cause of morbidity in children, including chronic malnutrition [3].

The original United Nations Millennium Development Goals included a target to reduce the mortality rate among children under five by two-thirds by 2015. Although tremendous progress has been made in reducing the rate by 47% to date [4], more work is urgently needed. Pursuing this target as part of the new Sustainable Development Goals [5] will require new approaches. These should include the wider use of oral rehydration solution (ORS) and expanded use of safe, effective, and affordable drugs such as antisecretory compounds [6] and antimicrobials targeting prominent causes of diarrheal disease in young children [7]. Further investment in research and development (R&D) focused on new drugs that are effective against some of the most important pathogens is also required.

The Global Enteric Multicenter Study (GEMS) was a multiyear study that analyzed moderate-to-severe diarrheal disease in children at seven sites across Africa and Asia using a case-control design [8]. Complementing the approach taken by GEMS, investigators from A Global Network for the Study of Malnutrition and Enteric Diseases (MAL-ED) conducted an equally ambitious birth cohort study over five years at eight community sites in Africa, Asia, and South America and assessed pathogen-specific burdens in diarrheal and nondiarrheal stool specimens [9].

Despite important differences in study design, both GEMS and MAL-ED call attention to the frequent and important role played by *Cryptosporidium* spp. (especially *C*. *hominis* and *C*. *parvum*) in causing morbidity and mortality among young children. In GEMS, *Cryptosporidium* spp. were identified as the second leading pathogen associated with moderate-to-severe diarrhea (MSD) in children under two years old and the leading pathogen associated with death in toddlers (ages 12 to 23 months) [8]. In the MAL-ED cohort, *Cryptosporidium* spp. were among the pathogens with the highest attributable burden of diarrhea among children one year old or younger and were also associated with persistent diarrhea [9]. The frequent or chronic insults to the gastrointestinal system by enteric pathogens including *Cryptosporidium*, alone or by coinfection, are thought to result in a condition known as environmental enteropathy, which manifests itself in malnutrition, stunting, perturbation of the gut microbiome, impaired cognitive development, diminished oral vaccine efficacy, and increased susceptibility to infections [10–13].

Although the diarrhea associated with *Cryptosporidium* infection can be life-threatening, it is only part of the problem. In a longitudinal study of slum dwellers in southern India, Ajjampur et al. [14] found that half of children were shedding oocysts well before or after episodes of cryptosporidial diarrhea, indicating the presence of asymptomatic infections. These asymptomatic infections are likely to contribute significantly to the long-term impact of cryptosporidiosis on malnutrition and stunted growth. This finding also has important implications for the control of *Cryptosporidium* and begs the question as to whether the problem should be addressed with a mass drug administration (MDA) strategy.

In considering the total effects of diarrheal disease in children—including cryptosporidiosis—we must also consider the tremendous financial burden borne by families with children experiencing multiple bouts of diarrheal disease each year. A study by Rheingans et al. [15] looked at African sites participating in GEMS and found that the total cost of treatment per episode of diarrhea ranged from US$6.01 in Mali to US$8.83 in Kenya, which is a huge amount in countries where more than one-third of the population lives below the international poverty line (of less than US$1.25 per day) and where total annual per capita health expenditure is US$53 and US$45, respectively [16–19].

Although *Cryptosporidium* was originally identified in 1907 [20], it was not until the late 1970s that its role as a serious, and sometimes lethal, human pathogen was elucidated by the careful work of Nime [21], Bird and Smith [22], Tzipori [23,24], and others [25,26]. In particular, the morbidity and potential mortality associated with cryptosporidiosis came into focus through the lens of the evolving HIV/AIDS epidemic in the 1980s [27]. Likewise, *Cryptosporidium* was demonstrated to be a common cause of diarrheal disease in children in Central Africa in the 1980s [28].

Despite the role of *Cryptosporidium* as an important human pathogen, there are currently few effective options for treatment. As reviewed by Checkley et al. [29], a variety of diagnostic methods exist, but they are best suited for epidemiological studies and are not routinely used in limited-resource settings. In addition, no vaccine is currently available to protect against *Cryptosporidium* infection and, based on the limited understanding of the *Cryptosporidium*-specific biology and challenges encountered in developing vaccines for other parasites, it is highly unlikely that a vaccine will be available within the next decade [29–31]. Moreover, although prevention for some enteric pathogens may be bolstered through programs focused on clean water and effective sanitation, such approaches will only go so far in preventing many cases of cryptosporidiosis, and person-to-person transmission further complicates the overall approach [32,33].

ORS-based treatment is a highly efficacious and cost-effective way to counteract the effects and relieve some of the symptoms associated with acute secretory diarrheas such as that caused by *Cryptosporidium*. However, use of ORS (particularly among children living in especially poor and rural areas) remains quite low, in part because treatment is labor-intensive and requires large volumes of ORS to be administered in order to rehydrate and then maintain hydration [34]. Furthermore, ORS has no role in the treatment of asymptomatic but insidious *Cryptosporidium* infections, which can only be addressed through drug treatment. Thus, to reduce the duration and impact of the *Cryptosporidium* infection, an antimicrobial drug is an important complement to ORS.

Only one drug, nitazoxanide (Alinia; Romark Laboratories, Tampa, Florida, United States), is currently approved by the US Food and Drug Administration (FDA) for treatment of *Cryptosporidium* infection. It is approved for use in children one year old or older [35]. The drug is available in many middle-income countries in Latin America, as well as in some low- and middle-income countries in Asia, including India and Bangladesh. Nonetheless, its actual use is limited, and empiric treatment with ORS alone remains the standard of care for diarrhea caused by *Cryptosporidium*.

Clinical studies have demonstrated the effectiveness of nitazoxanide against *Cryptosporidium* in otherwise healthy individuals. In addition, clinical studies have provided evidence suggesting the drug’s activity against rotavirus, helminths, and other enteric protozoan parasites [36–38]. Thus, there are sufficient reasons for considering wider use of this drug, even as a candidate for use in MDA programs as proposed by Hotez [39] and others.

The drug’s efficacy profile is variable, with cure rates reported to range from more than 80% in otherwise healthy adults to only 56% in malnourished children [40]. One significant shortcoming of nitazoxanide is that it does not appear to be beneficial in individuals who are immunocompromised [41] and is less effective in children who are malnourished—a key limitation given the prevalence of immunosuppression in children who are HIV-positive and/or malnourished.

We support an expanded global role for nitazoxanide in addressing cryptosporidiosis in children under five years of age if the global community is to meet established goals for reducing childhood mortality. We intend to investigate the possibility of lowering the approved age for treatment by testing the drug’s safety and effectiveness in children between six and 12 months of age, to explore rational combinations with other drugs (see Table 1), and to have the drug included on the World Health Organization’s Model List of Essential Medicines.

**Table 1:** Drugs and drug candidates for treating *Cryptosporidium* infection in young children.

Compound	Development stage	Activity against *Cryptosporidium*	References
Nitazoxanide	FDA approved for *Cryptosporidium*, launched	Clinical	[40,44–46]
Nitazoxanide + Azithromycin	Proposed combination therapy	Anecdotal clinical evidence	[47–48]
Pyrvinium pamoate	Repurposing candidate (FDA approved for pinworm infections)	Animal model	[49]
Pitavastatin	Repurposing candidate (FDA approved for hypercholesterolemia)	In vitro only (yet to be tested in an animal model)	[50]
Auranofin	Repurposing candidate (FDA approved for rheumatoid arthritis)	In vitro only (yet to be tested in an animal model)	[51]
Calcium-dependent protein kinase 1 inhibitors	Preclinical	Animal model	[52]
Inosine 5’- monophosphate dehydrogenase (IMPDH) inhibitors	Preclinical	Animal model	[53]
Fatty acyl-CoA synthetase inhibitor Triacsin C	Preclinical	Animal model	[54]
Oleylphosphocholine	Preclinical	Animal model	[55]
Medicines for Malaria Venture (MMV) Malaria Box compounds	Preclinical	In vitro only (yet to be tested in an animal model)	[56]

**Fig 1:**
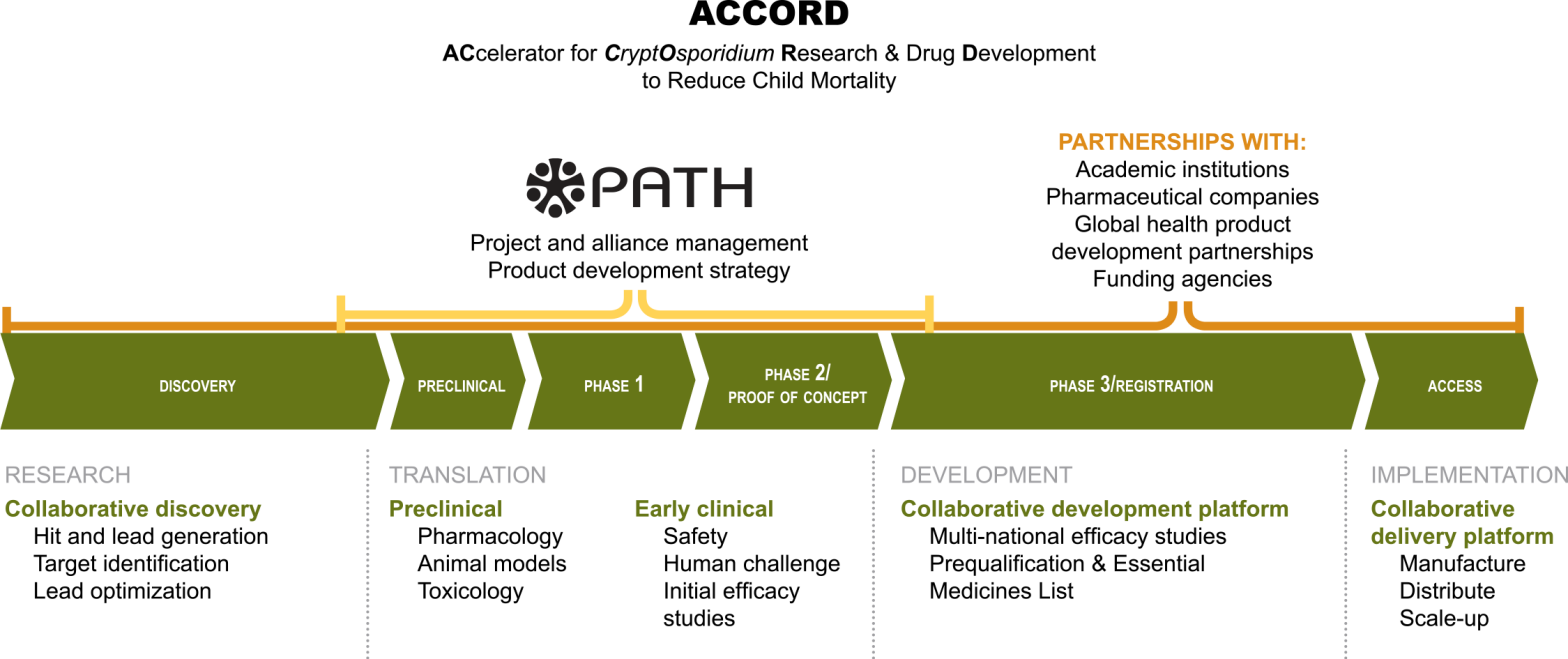
Proposed ACCORD (ACcelerator for *CryptOsporidium* Research & Drug Development to Reduce Child Mortality) structure and function. The ACCORD approach is an integrated partnership among pharmaceutical companies, research institutions, product development partnerships, and funders to accelerate research and development of needed *Cryptosporidium* drugs to reduce child mortality.

While the wider use of nitazoxanide would help relieve the burden of *Cryptosporidium* infection, we also see an urgent need for new drugs that provide an alternative to nitazoxanide and address its shortcomings. Several groups have identified existing drugs in clinical use for other indications that have the potential to be repurposed for the treatment of *Cryptosporidium* infection (Table 1). In addition, there are a number of preclinical leads in the pipeline, but more are needed. Now is the time to develop a bold, integrated program to accelerate funding, research, and development of needed *Cryptosporidium* drugs. Taking as a guiding model the Tuberculosis Drug Accelerator established by the Bill & Melinda Gates Foundation, we propose an approach that we call ACCORD (ACcelerator for *CryptOsporidium* Research & Drug Development to Reduce Child Mortality) to help accelerate the development of new therapeutics for *Cryptosporidium* (Fig 1).

ACCORD will be successful only with investments and commitments from pharmaceutical companies, private foundations, governments, academic institutions, and nongovernmental organizations. In 2014, the authors of the G-FINDER report meticulously studied and analyzed global diarrheal disease R&D funding [42]. The authors found that, in 2013, investment specific to *Cryptosporidium* was limited to US$2.7 million in basic research and US$1.7 million in R&D focused on drugs. This is a paltry sum when compared to the amount spent on R&D for tuberculosis drugs (US$248 million) or a rotavirus vaccine (more than US$200 million) [43]. We believe that at least US$10 million of new funding per year is required for the next decade in order to develop the next generation of drugs. With this increased investment in drug development for diarrheal disease, and through collaborative partnerships across sectors, we will be able to align and accelerate the efforts needed to achieve further progress in meeting global targets for reductions in child mortality.
